# 1-(4-Carb­oxy­butan-2-yl­idene)-4-phenyl­thio­semicarbazide

**DOI:** 10.1107/S1600536812023793

**Published:** 2012-05-31

**Authors:** Rafael Mendoza-Meroño, Laura Menéndez-Taboada, Santiago García-Granda

**Affiliations:** a Departamento de Química Física y Analítica, Facultad de Química, Universidad de Oviedo - CINN, C/ Julián Clavería, 8, 33006 Oviedo, Spain

## Abstract

The mol­ecule of the title compound, C_12_H_15_N_3_O_2_S, which belongs to the family of thio­semicarbazones, containing an acid group, adopts a semi-closed conformation with an intramolecular N—H⋯N hydrogen bond. In the crystal, molecules are linked by strong N—H⋯O and O—H⋯S hydrogen bonds between the acid group and thiosemicarbazone unit, with one additional intermolecular hydrogen C—H⋯O interaction. These three interactions form *R*_2_^2^(8) and a *R*_2_^1^(7) rings and the molecules related by the *c*-glide plane are linked into a zigzag chain along [001].

## Related literature

For related compounds and their biological activity, see: Ng (1992 ▶); Papageorgiou *et al.* (1997 ▶); Du *et al.* (2002 ▶). For a description of the Cambridge Crystallographic Database, see: Allen (2002 ▶). For hydrogen-bond motifs, see: Bernstein *et al.* (1995 ▶).
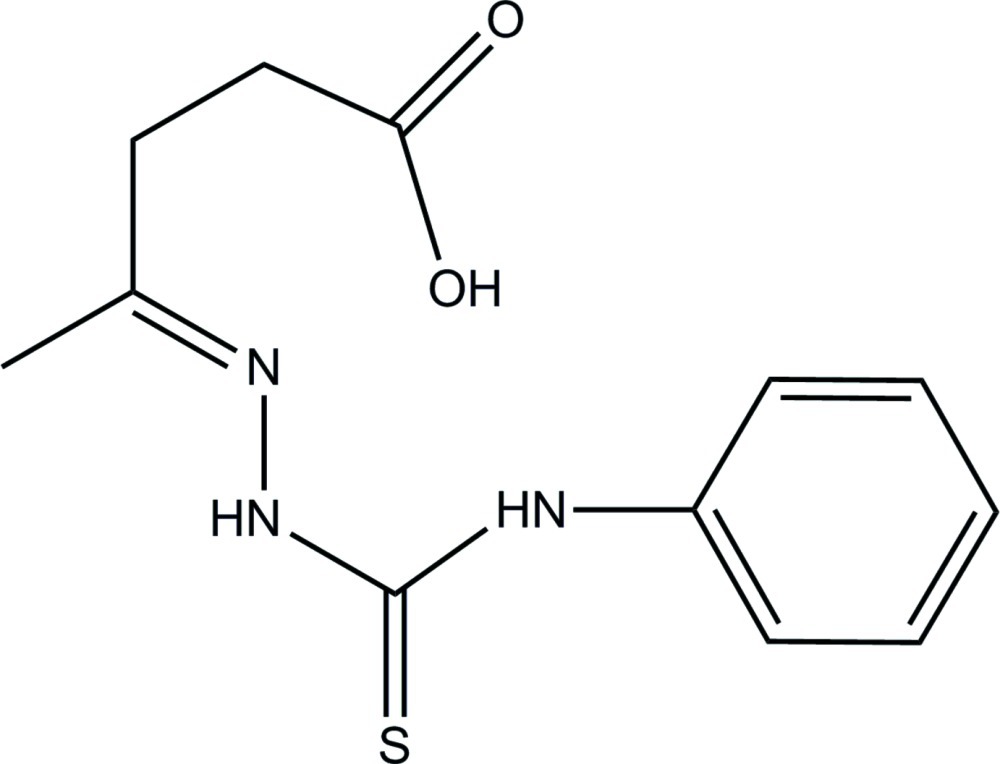


## Experimental

### 

#### Crystal data

C_12_H_15_N_3_O_2_S
*M* *_r_* = 265.33
Monoclinic, 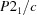

*a* = 11.2812 (4) Å
*b* = 9.3450 (4) Å
*c* = 13.4120 (5) Å
β = 104.176 (3)°
*V* = 1370.87 (9) Å^3^
*Z* = 4
Cu *K*α radiation
μ = 2.10 mm^−1^
*T* = 293 K
0.26 × 0.18 × 0.12 mm

#### Data collection

Oxford Diffraction Xcalibur Ruby Gemini diffractometer
Absorption correction: multi-scan (*CrysAlis RED*; Oxford Diffraction, 2010 ▶)*T*_min_ = 0.907, *T*_max_ = 1.000
12283 measured reflections
2601 independent reflections
2294 reflections with *I* > 2σ(*I*)
*R*_int_ = 0.027

#### Refinement

*R*[*F* ^2^ > 2σ(*F* ^2^)] = 0.040
*wR*(*F* ^2^) = 0.114
*S* = 1.05
2601 reflections
191 parameters
H atoms treated by a mixture of independent and constrained refinement
Δρ_max_ = 0.30 e Å^−3^
Δρ_min_ = −0.28 e Å^−3^

### 

Data collection: *CrysAlis CCD* (Oxford Diffraction, 2010 ▶); cell refinement: *CrysAlis RED* (Oxford Diffraction, 2010 ▶); data reduction: *CrysAlis RED*; program(s) used to solve structure: *SIR92* (Altomare *et al.*, 1994 ▶); program(s) used to refine structure: *SHELXL97* (Sheldrick, 2008 ▶); molecular graphics: *ORTEP-3 for Windows* (Farrugia, 1997 ▶) and *Mercury* (Macrae *et al.*, 2008 ▶); software used to prepare material for publication: *WinGX* publication routines (Farrugia, 1999 ▶), *PLATON* (Spek, 2003 ▶), *PARST95* (Nardelli, 1995 ▶) and *publCIF* (Westrip, 2010 ▶).

## Supplementary Material

Crystal structure: contains datablock(s) global, I. DOI: 10.1107/S1600536812023793/ds2195sup1.cif

Structure factors: contains datablock(s) I. DOI: 10.1107/S1600536812023793/ds2195Isup2.hkl

Supplementary material file. DOI: 10.1107/S1600536812023793/ds2195Isup3.cml

Additional supplementary materials:  crystallographic information; 3D view; checkCIF report

## Figures and Tables

**Table 1:** Hydrogen-bond geometry (Å, °)

*D*—H⋯*A*	*D*—H	H⋯*A*	*D*⋯*A*	*D*—H⋯*A*
N3—H3*N*⋯N1	0.81 (2)	2.06 (2)	2.547 (2)	118.1 (19)
N2—H2*N*⋯O2^i^	0.83 (2)	2.19 (2)	3.013 (2)	174 (2)
C5—H5*A*⋯O2^i^	0.96	2.23	3.184 (3)	174
O1—H1*O*⋯S1^ii^	0.92 (3)	2.24 (3)	3.1600 (17)	174 (3)

Symmetry codes: (i) 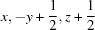; (ii) 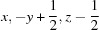.

**Fig. 1.**
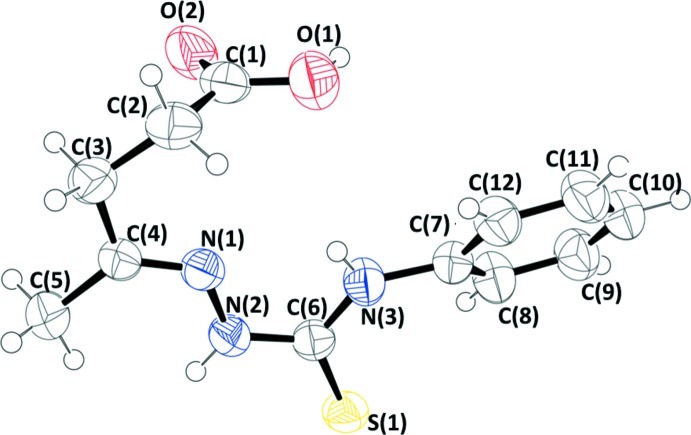
A view of the molecular structure of the title molecule showing the atom-numbering scheme. Displacement ellipsoids are drawn at the 50% probability level.

**Fig. 2.**
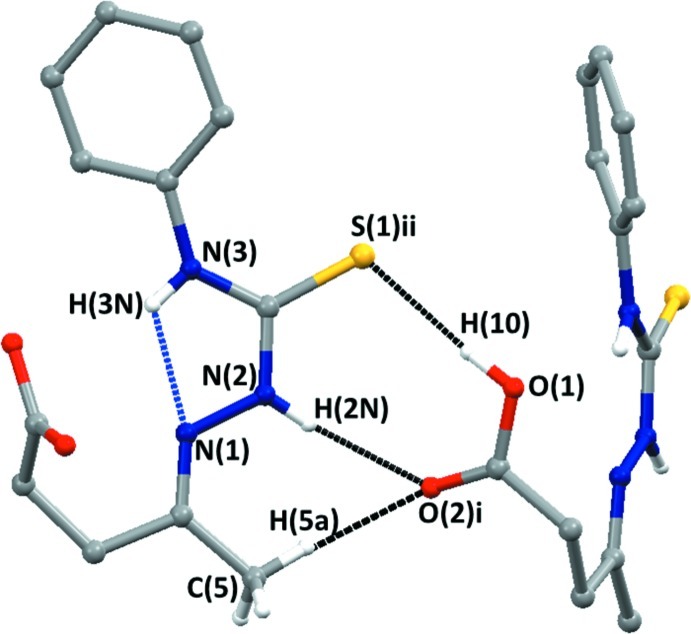
Principal intermolecular and intramolecular hydrogen bonds. H atoms not involved in hydrogen bonding have been omitted for clarity.

**Fig. 3.**
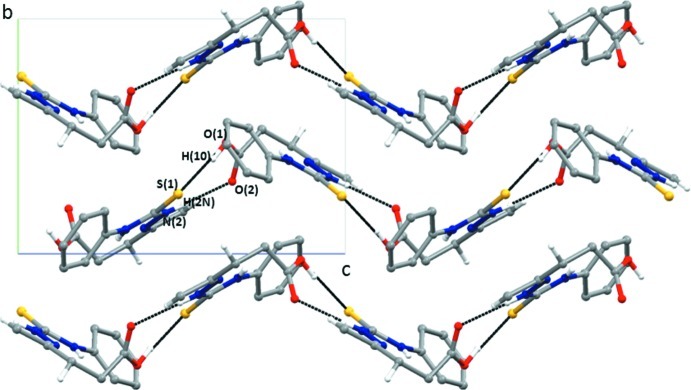
Packing diagram viewed along the c axis , showing the zig-zag chains. Hydrogen bonds are indicated by dashed lines.

## supplementary crystallographic information

### Comment

Thiosemicarbazones have been extensively studied due to their wide range of actual or potential medical applications which include notably antiparasital (Du *et al.*, 2002) and antitumor activities (Papageorgiou *et al.* 1997). In this work we have synthesized and crystallized a new thiosemicarbazone (I). Fig(1)

The molecule in the crystal adopt a *semi-closed conformation*, similiar to the structure reported by Ng (1992) [CSDRefcode: JUBMAU] .The values of distances N–N length (1.380 (2) Å.) and the dihedral angle C═ N—N—C (177.2 (2) °) are similar to those found in CSD (Allen, 2002) for thiosemicarbazone systems [selected 371 hits, average distance N—N is 1.374Å and mean dihedral angle is 178.21 °].

In the crystal packing the strong interactions are stablished between *acid group* and *thiosemicarbazone moiety* through N(2)—H(2N)···O(2) and O(1)—H(1O)···S(1). There is one additional intermolecular hydrogen C(5)—H(5a)···O(2) interaction. These three interactions form a R^2^_2_(8) and a R^1^_2_(7) rings (Bernstein *et al.*, 1995) as shown in Fig (2). The molecules linked in this way form a *zig-zag* chain crystallograhycally related by *c-glide* plane shown in Fig (3). An intramolecular N(3)—H(3N)···N(1) hydrogen bond is also present. (Table 1).

### Experimental

A solution of levulinic acid (1.1612 g, 0.01 mol) and 4-phenylsemicarbazide (1.6723 g, 0.01 mol) in absolute methanol (50 ml) was refluxed for 1 h in the presence of *p*-toluenesulfonic acid as catalyst, with continuous stirring. On cooling to room temperature the precipitate was filtered off, washed with copious cold methanol and dried in air. White single crystals of compound (I) were obtained after recrystallization from a solution in methanol.

### Refinement

The NH ,CH_2_ and OH H-atoms were found in difference Fourier maps and were freely refined: N2—H = 0.83 (2) Å, N3—H = 0.81 (2) Å , C3—H=0.97 (2) Å, C2—H(2A)=0.94 (2) Å, C2—H(2B)=0.98 (2) Å and O(2)—H=0.92 (3) Å. All other C-bound H-atoms were included in calculated positions and treated as riding atoms: C—H = 0.93 Å for aromatic CH with U_iso_(H) = 1.2 × U_eq_(C) and CH_3_ with U_iso_(H) = 1.5 × U_eq_(C). At the end of the refinement the highest peak in the electron density was 0.301 eÅ ^-3^, while the deepest hole was -0.283 eÅ ^-3^.

### Crystal data

**Table:**

C_12_H_15_N_3_O_2_S	*F*(000) = 560
*M**_r_* = 265.33	*D*_x_ = 1.286 Mg m^−^^3^
Monoclinic, *P*2_1_/*c*	Melting point: 437 K
Hall symbol: -P 2ybc	Cu *K*α radiation, λ = 1.54180 Å
*a* = 11.2812 (4) Å	Cell parameters from 6997 reflections
*b* = 9.3450 (4) Å	θ = 3.4–70.4°
*c* = 13.4120 (5) Å	µ = 2.10 mm^−^^1^
β = 104.176 (3)°	*T* = 293 K
*V* = 1370.87 (9) Å^3^	Blocks, white
*Z* = 4	0.26 × 0.18 × 0.12 mm

### Data collection

**Table:**

Oxford Diffraction Xcalibur Ruby Gemini diffractometer	2601 independent reflections
Radiation source: Enhance (Cu) X-ray Source	2294 reflections with *I* > 2σ(*I*)
Graphite monochromator	*R*_int_ = 0.027
Detector resolution: 10.2673 pixels mm^-1^	θ_max_ = 70.5°, θ_min_ = 4.0°
ω scans	*h* = −13→12
Absorption correction: multi-scan (*CrysAlis RED*; Oxford Diffraction, 2010) Empirical absorption correction using spherical harmonics, implemented in SCALE3 ABSPACK scaling algorithm.	*k* = −11→10
*T*_min_ = 0.907, *T*_max_ = 1.000	*l* = −16→16
12283 measured reflections	

### Refinement

**Table:**

Refinement on *F*^2^	Primary atom site location: structure-invariant direct methods
Least-squares matrix: full	Secondary atom site location: difference Fourier map
*R*[*F*^2^ > 2σ(*F*^2^)] = 0.040	Hydrogen site location: inferred from neighbouring sites
*wR*(*F*^2^) = 0.114	H atoms treated by a mixture of independent and constrained refinement
*S* = 1.05	*w* = 1/[σ^2^(*F*_o_^2^) + (0.0645*P*)^2^ + 0.3083*P*] where *P* = (*F*_o_^2^ + 2*F*_c_^2^)/3
2601 reflections	(Δ/σ)_max_ < 0.001
191 parameters	Δρ_max_ = 0.30 e Å^−^^3^
0 restraints	Δρ_min_ = −0.28 e Å^−^^3^

### Special details

**Table:**

Geometry. All esds (except the esd in the dihedral angle between two l.s. planes) are estimated using the full covariance matrix. The cell esds are taken into account individually in the estimation of esds in distances, angles and torsion angles; correlations between esds in cell parameters are only used when they are defined by crystal symmetry. An approximate (isotropic) treatment of cell esds is used for estimating esds involving l.s. planes.
Refinement. Refinement of F^2^ against ALL reflections. The weighted R-factor wR and goodness of fit S are based on F^2^, conventional R-factors R are based on F, with F set to zero for negative F^2^. The threshold expression of F^2^ > 2sigma(F^2^) is used only for calculating R-factors(gt) etc. and is not relevant to the choice of reflections for refinement. R-factors based on F^2^ are statistically about twice as large as those based on F, and R- factors based on ALL data will be even larger.

### Fractional atomic coordinates and isotropic or equivalent isotropic displacement parameters (Å^2^)

**Table:**

	*x*	*y*	*z*	*U*_iso_*/*U*_eq_	
S1	0.78464 (4)	0.25421 (5)	0.48897 (3)	0.05613 (18)	
N2	0.99203 (13)	0.17312 (16)	0.44863 (11)	0.0480 (3)	
N1	1.05662 (12)	0.10898 (15)	0.38565 (10)	0.0461 (3)	
O1	0.95660 (14)	0.02621 (17)	0.13593 (12)	0.0715 (4)	
N3	0.82411 (14)	0.10334 (16)	0.32848 (12)	0.0519 (4)	
O2	1.10103 (15)	0.19167 (18)	0.15461 (11)	0.0776 (5)	
C4	1.17284 (14)	0.11152 (18)	0.40985 (12)	0.0470 (4)	
C7	0.70327 (15)	0.08424 (18)	0.26779 (12)	0.0479 (4)	
C6	0.86830 (14)	0.17285 (17)	0.41730 (12)	0.0452 (4)	
C5	1.25209 (18)	0.1801 (3)	0.50355 (15)	0.0701 (6)	
H5A	1.2016	0.2207	0.5445	0.105*	
H5B	1.3002	0.2542	0.4832	0.105*	
H5C	1.3052	0.1094	0.5431	0.105*	
C3	1.23533 (17)	0.0414 (2)	0.33614 (14)	0.0549 (4)	
C10	0.47481 (18)	0.0357 (2)	0.13805 (15)	0.0642 (5)	
H10	0.3979	0.0192	0.0949	0.077*	
C8	0.60898 (17)	0.1802 (2)	0.26478 (15)	0.0610 (5)	
H8	0.6220	0.2617	0.3058	0.073*	
C2	1.14856 (19)	−0.0293 (2)	0.24553 (15)	0.0587 (5)	
C1	1.06851 (18)	0.0751 (2)	0.17557 (13)	0.0563 (4)	
C12	0.68317 (17)	−0.0348 (2)	0.20479 (14)	0.0567 (4)	
H12	0.7464	−0.0991	0.2059	0.068*	
C9	0.49515 (18)	0.1538 (2)	0.20007 (16)	0.0662 (5)	
H9	0.4314	0.2175	0.1988	0.079*	
C11	0.56902 (19)	−0.0583 (2)	0.14005 (15)	0.0657 (5)	
H11	0.5559	−0.1384	0.0976	0.079*	
H2A	1.1951 (19)	−0.075 (2)	0.2050 (16)	0.064 (6)*	
H2B	1.097 (2)	−0.101 (2)	0.2673 (17)	0.073 (6)*	
H3B	1.2903 (19)	−0.032 (2)	0.3704 (16)	0.065 (6)*	
H3A	1.2844 (18)	0.113 (2)	0.3127 (15)	0.059 (5)*	
H2N	1.027 (2)	0.208 (2)	0.5052 (18)	0.064 (6)*	
H3N	0.879 (2)	0.068 (2)	0.3071 (17)	0.063 (6)*	
H1O	0.910 (3)	0.096 (3)	0.096 (2)	0.108 (10)*	

### Atomic displacement parameters (Å^2^)

**Table:**

	*U*^11^	*U*^22^	*U*^33^	*U*^12^	*U*^13^	*U*^23^
S1	0.0539 (3)	0.0688 (3)	0.0476 (3)	0.00688 (19)	0.0162 (2)	−0.00087 (18)
N2	0.0457 (7)	0.0563 (8)	0.0410 (7)	−0.0005 (6)	0.0085 (6)	−0.0048 (6)
N1	0.0467 (7)	0.0504 (8)	0.0409 (7)	0.0020 (6)	0.0098 (6)	−0.0004 (6)
O1	0.0667 (9)	0.0708 (9)	0.0701 (9)	−0.0106 (7)	0.0035 (7)	0.0069 (7)
N3	0.0431 (7)	0.0569 (9)	0.0541 (8)	0.0008 (6)	0.0089 (6)	−0.0107 (7)
O2	0.0800 (9)	0.0864 (10)	0.0576 (8)	−0.0231 (8)	0.0000 (7)	0.0237 (7)
C4	0.0462 (8)	0.0525 (9)	0.0412 (8)	−0.0028 (7)	0.0082 (7)	0.0048 (7)
C7	0.0449 (8)	0.0520 (9)	0.0460 (8)	−0.0054 (7)	0.0094 (7)	0.0010 (7)
C6	0.0478 (8)	0.0430 (8)	0.0445 (8)	0.0002 (6)	0.0108 (7)	0.0046 (6)
C5	0.0507 (10)	0.1051 (17)	0.0524 (10)	−0.0135 (11)	0.0089 (8)	−0.0109 (10)
C3	0.0501 (9)	0.0641 (11)	0.0519 (10)	0.0064 (8)	0.0155 (8)	0.0066 (8)
C10	0.0520 (10)	0.0791 (13)	0.0556 (11)	−0.0102 (9)	0.0019 (8)	−0.0004 (9)
C8	0.0509 (10)	0.0628 (11)	0.0649 (11)	0.0019 (8)	0.0055 (8)	−0.0107 (9)
C2	0.0680 (12)	0.0591 (11)	0.0523 (10)	0.0051 (9)	0.0209 (9)	−0.0045 (8)
C1	0.0647 (11)	0.0649 (11)	0.0406 (8)	−0.0051 (9)	0.0153 (8)	−0.0021 (8)
C12	0.0564 (10)	0.0564 (10)	0.0551 (10)	0.0000 (8)	0.0095 (8)	−0.0052 (8)
C9	0.0504 (10)	0.0776 (13)	0.0657 (12)	0.0048 (9)	0.0049 (9)	−0.0022 (10)
C11	0.0677 (12)	0.0672 (12)	0.0569 (11)	−0.0104 (9)	0.0052 (9)	−0.0117 (9)

### Geometric parameters (Å, º)

**Table:**

S1—C6	1.6843 (17)	C5—H5C	0.9600
N2—C6	1.356 (2)	C3—C2	1.513 (3)
N2—N1	1.3802 (19)	C3—H3B	0.97 (2)
N2—H2N	0.83 (2)	C3—H3A	0.97 (2)
N1—C4	1.271 (2)	C10—C9	1.367 (3)
O1—C1	1.325 (2)	C10—C11	1.374 (3)
O1—H1O	0.92 (3)	C10—H10	0.9300
N3—C6	1.341 (2)	C8—C9	1.385 (3)
N3—C7	1.418 (2)	C8—H8	0.9300
N3—H3N	0.81 (2)	C2—C1	1.494 (3)
O2—C1	1.205 (2)	C2—H2A	0.94 (2)
C4—C5	1.496 (2)	C2—H2B	0.98 (2)
C4—C3	1.498 (2)	C12—C11	1.383 (3)
C7—C12	1.381 (3)	C12—H12	0.9300
C7—C8	1.384 (3)	C9—H9	0.9300
C5—H5A	0.9600	C11—H11	0.9300
C5—H5B	0.9600		
			
C6—N2—N1	117.86 (14)	C2—C3—H3A	110.4 (12)
C6—N2—H2N	120.3 (15)	H3B—C3—H3A	107.1 (17)
N1—N2—H2N	121.8 (15)	C9—C10—C11	119.38 (18)
C4—N1—N2	120.11 (14)	C9—C10—H10	120.3
C1—O1—H1O	110.1 (18)	C11—C10—H10	120.3
C6—N3—C7	131.84 (16)	C7—C8—C9	119.39 (18)
C6—N3—H3N	111.3 (15)	C7—C8—H8	120.3
C7—N3—H3N	116.9 (15)	C9—C8—H8	120.3
N1—C4—C5	126.09 (16)	C1—C2—C3	113.05 (16)
N1—C4—C3	116.48 (15)	C1—C2—H2A	105.5 (13)
C5—C4—C3	117.42 (15)	C3—C2—H2A	108.5 (12)
C12—C7—C8	119.60 (16)	C1—C2—H2B	108.7 (13)
C12—C7—N3	116.31 (16)	C3—C2—H2B	112.0 (13)
C8—C7—N3	124.00 (16)	H2A—C2—H2B	108.9 (18)
N3—C6—N2	114.00 (15)	O2—C1—O1	122.19 (18)
N3—C6—S1	125.96 (13)	O2—C1—C2	124.43 (18)
N2—C6—S1	120.04 (12)	O1—C1—C2	113.37 (17)
C4—C5—H5A	109.5	C7—C12—C11	120.02 (18)
C4—C5—H5B	109.5	C7—C12—H12	120.0
H5A—C5—H5B	109.5	C11—C12—H12	120.0
C4—C5—H5C	109.5	C10—C9—C8	121.13 (19)
H5A—C5—H5C	109.5	C10—C9—H9	119.4
H5B—C5—H5C	109.5	C8—C9—H9	119.4
C4—C3—C2	113.87 (15)	C10—C11—C12	120.47 (19)
C4—C3—H3B	110.5 (12)	C10—C11—H11	119.8
C2—C3—H3B	106.5 (13)	C12—C11—H11	119.8
C4—C3—H3A	108.3 (12)		
			
C6—N2—N1—C4	177.24 (15)	C12—C7—C8—C9	1.3 (3)
N2—N1—C4—C5	−0.2 (3)	N3—C7—C8—C9	177.77 (18)
N2—N1—C4—C3	−179.00 (15)	C4—C3—C2—C1	66.2 (2)
C6—N3—C7—C12	−155.39 (18)	C3—C2—C1—O2	37.4 (3)
C6—N3—C7—C8	28.0 (3)	C3—C2—C1—O1	−143.94 (17)
C7—N3—C6—N2	−176.52 (17)	C8—C7—C12—C11	−0.7 (3)
C7—N3—C6—S1	4.0 (3)	N3—C7—C12—C11	−177.45 (17)
N1—N2—C6—N3	2.5 (2)	C7—C8—C9—C10	−1.0 (3)
N1—N2—C6—S1	−177.98 (11)	C9—C10—C11—C12	0.6 (3)
N1—C4—C3—C2	−3.1 (2)	C7—C12—C11—C10	−0.2 (3)
C5—C4—C3—C2	178.08 (18)		

### Hydrogen-bond geometry (Å, º)

**Table:**

*D*—H···*A*	*D*—H	H···*A*	*D*···*A*	*D*—H···*A*
N3—H3*N*···N1	0.81 (2)	2.06 (2)	2.547 (2)	118.1 (19)
N2—H2*N*···O2^i^	0.83 (2)	2.19 (2)	3.013 (2)	174 (2)
C5—H5*A*···O2^i^	0.96	2.23	3.184 (3)	174
O1—H1*O*···S1^ii^	0.92 (3)	2.24 (3)	3.1600 (17)	174 (3)

Symmetry codes: (i) *x*, −*y*+1/2, *z*+1/2; (ii) *x*, −*y*+1/2, *z*−1/2.
